# Health information technology use among foreign-born adults of Middle Eastern and North African decent in the United States

**DOI:** 10.21203/rs.3.rs-3491745/v1

**Published:** 2023-10-26

**Authors:** Alexandra Smith, Tiffany Kindratt

**Affiliations:** University of Texas at Arlington; University of Texas at Arlington

**Keywords:** Health information technology, Middle Eastern and North African, Arab American, National Health Interview Survey

## Abstract

Health information technology (HIT) use among foreign-born adults of Middle Eastern and North African (MENA) descent living in America is an understudied population. They are currently categorized as “White” in the United States (US) on federal forms. The purpose was to uncover the prevalence of HIT use among MENA immigrants compared to US- and foreign-born White adults before and after adjusting for other factors. The 2011–2018 National Health Interview Survey data (n = 161,613; ages 18 + years) was analyzed. HIT uses evaluated were searching for health information, filling prescriptions, scheduling appointments, and communicating with healthcare providers via email (last 12 months). Crude and multivariable logistic regression models were used to estimate the odds of each HIT use, any HIT use, and all HIT uses before and after adjustment. The most common HIT use was looking up health information, with 46.4% of foreign-born adults of MENA, 47.8% of foreign-born White, and 51.2% of US-born White adults reporting its use (p = .0079). Foreign-born adults of MENA descent had lower odds (OR = 0.64; 95%CI = 0.56–0.74) of reporting any HIT use, but no difference in reporting all HIT uses compared to US-born White adults in adjusted models. This is the first study to explore HIT use among Americans of MENA descent. Patterns of HIT use among adults of MENA descent differ from White adults. Results contribute to growing body of literature showing the health of Americans of MENA descent differs from White Americans. A separate racial/ethnic identifier is needed to better capture HIT uses among populations of MENA descent.

## BACKGROUND

The use of health information technology (HIT) has become progressively popular as development in technology has advanced. HIT use involves accessing the internet to seek healthcare information, consulting with healthcare professionals, and searching other health-related inquiries. Individuals and healthcare providers have increased technology use as a mode of communication and education to seek and provide information outside of office visits.^1^ With an advancement in HIT use, there is interest about who is using it, how often, and for what means. Previous studies have sought to identify characteristics of individuals using HIT and potential purposes. Manganello (2017) found regardless of self-report health understanding there was an increase in HIT use.^2^ Gandrakota (2021) found similar results in their study of HIT use from the years 2012 to 2018. They also found sociodemographic differences; older individuals and individuals that identified as a racial or ethnic minority used HIT less compared to Non-Hispanic White adults (hereafter, White).^3^

On a national level, studies have been conducted to examine differences in HIT use based on sociodemographic factors and found that HIT use differs by age, gender, sexual orientation, education attainment, nativity status, and race/ethnicity. Younger adults, women, sexual minority groups, adults living with chronic conditions, and racial/ethnic minority groups are more likely to report HIT use compared to their peers.^4–7^ Black, Hispanic, and Asian adults are less likely to report any HIT use compared to White adults.^8,9^ Regarding nativity status, other research has reported that foreign-born Hispanic adults are less likely to report HIT use compared to US-born Hispanic and US-born White adults.^10^ Research has been conducted to evaluate HIT use as a potential predictor of preventive service utilization. Kindratt and colleagues (2019) evaluated how HIT use was associated with influenza vaccine uptake and found that any HIT users were more likely to receive an influenza vaccine compared to those who did not report any HIT use.^11^ Other studies have reported that HIT users were more likely to report receiving recommended cancer screenings, pneumonia vaccines, and HIV testing compared to adults who reported zero HIT use.^11–15^

### Theoretical/Conceptual Framework

Previous studies evaluating HIT use fail to account for the disparities that exist among populations who lack representation on a national level, specifically adults of Middle Eastern and North African (MENA) descent. The lack of studies on MENA Americans is because this population is defined as “White” by the federal Office of Management and Budget. The White population comprises individuals who were born in or trace their heritage to countries located in Europe, the Middle East, and North Africa. Adults of MENA descent are considered White on surveys such as the National Health Interview Survey (NHIS) and the US Census Bureau. Despite this classification, research studies have found that the lived experiences and health of this population differs from White adults.^16^ This can lead to a lack of representation, support, and federal funding. The MENA community is not always perceived as “White” by society and can face discrimination due to race, appearance, and cultural practices. Awad and colleagues (2019) noted that MENA individuals can experience trauma and discrimination after major events like 9/11, and major political changes.^17^ Regarding health, studies have reported that MENA adults are more likely to report psychological health concerns,^18,19^ comorbid diabetes,^20^ and disabilities,^21–24^ less likely to report receiving immunizations and cancer screenings,^25–27^ less likely to receive a diagnosis for dementia,^28^ and less likely to receive recommended follow-up care compared to White adults. ^29,30^ Although studies have found that HIT use may contribute to improvements in healthcare utilization and management of the chronic conditions that disproportionately affect this population, patterns of HIT use among the MENA population remains unknown.

To the authors’ knowledge, there are no current studies that analyze HIT use among foreign-born adults of MENA descent. Our aim was to estimate and compare the prevalence of HIT use among foreign-born adults of MENA descent compared to US- and foreign-born White adults before and after adjusting for covariates.

## METHODS

### Data Source and Participants

Secondary data from the 2011–2018 NHIS were analyzed. The NHIS conducted by the National Center for Health Statistics collects health information about the US population. Data are collected in a face-to-face interview at the home of the participants throughout the year. Questions are asked about the family, one sample child (17 years or younger), and one sample adult (18 years or older) at each household. Starting in the year 2011 questions were asked to the sample adult about HIT use. Our sample included 161,613 participants aged 18 + years (n = 1,264 foreign-born MENA, n = 4,516 foreign-born White, and n = 155,833 US-born White). Data were analyzed using the complex sample design features in SAS 9.4.

### Variables

#### Independent Variable

The independent variables were created from NHIS question cards accessing race, ethnicity, and region of birth. Participants selected their race (White, Black, American Indian/Alaskan Native, Asian, Other) and identified if they were Hispanic or Latino. Participants were asked their birthplace (one of the 50 states, Washington DC, military base overseas, or US territory). Individuals not born in the US they were asked “in what country were you born?” All responses were divided into 10 world regions (US, Europe, Russia, Middle East, Africa, and others). Individuals born in the “Middle East” or “Africa” were categorized as foreign-born adults of MENA descent and compared to US- and foreign-born White (from Europe or Russia).

#### Dependent Variable

From 2011–2018, the NHIS consistently measured HIT use in a series of four questions. The questions included assessed whether the participant (no, yes) used the internet during the past 12 months to look up health information, fill a prescription, scheduled medical appointments, or communicate with a healthcare provider by email.^31^” Questions were analyzed individually and collectively as “any” HIT use and “all” HIT use based on previous studies ^11,32^.

### Covariates

Covariates included age (18–44, 45–64, 65 + years), sex (male, female), highest level of education (less than high school, high school diploma or GED, some college or associate degree, bachelor’s degree or higher) and chronic conditions. Chronic conditions were evaluated in two ways. Diabetes, heart disease, cancer, and asthma were identified as four of the top ten leading causes of death in the US.^33^ Studies have found that that the burden of these chronic conditions differs from White adults^20,30,34^ and people with chronic conditions (e.g., diabetes) are more and less (e.g., cardiovascular disease risk) likely to report HIT use compared to other adults.^35,36^ Participants were asked whether they have ever been told by a healthcare provider that they had diabetes, coronary heart disease, cancer, or asthma.^31^ A dichotomous variable (no, yes) was created for each chronic condition. Then, a combined variable was created to indicate whether participants had one or more (no, yes) of the top chronic conditions.

### Institutional Review Board

This study used secondary data that did not include any identifiable information and were publicly available. Therefore, our institutional review board deemed this study not subject for review or approval.

## RESULTS

Table 1 provides the sociodemographics, top chronic conditions, and HIT uses. The MENA population was younger, with 50.1% of foreign-born adults of MENA descent ages 18–44 years compared to 38.3% of foreign-born White and 41.6% of US-born White adults (p = .0001). A greater amount of MENA adults were men (54.1%) compared to US- and foreign-born White adults (48.5% and 45.6%, respectively) (p = .0111). More foreign-born MENA adults had a bachelor’s degrees or higher level of education (50.2%) compared to US- and foreign-born White counterparts (32.5% and 42.3%, respectively) (p < .0001). Fewer foreign-born adults of MENA descent reported any top chronic conditions (21.0%) compared to foreign-born (23.8%) and US-born White (31.7%) adults (p < .0001). Fewer foreign-born adults of MENA descent reported all HIT uses (48.9%) compared to US- (53.6%) and foreign-born (50.3%) White adults (p = .0102). Specifically, foreign-born adults of MENA descent were less likely to report looking up health information and filling prescriptions online compared to US- and foreign-born White adults (both p’s < .05) yet were more likely to schedule appointments with a healthcare provider online compared to other groups (p = .0274). Only 2.9% of foreign-born adults of MENA descent reported all HIT uses compared to 2.2% of foreign-born and 2.5% of US-born White adults.

Table 2 provides associations between race, ethnicity and nativity and any HIT use. Foreign-born adults of MENA descent had 17% lower odds (95%CI= 0.70–0.92) of any HIT use compared to US-born White adults. Results remained statistically significant when adjusted for age, sex, and highest level of education, and any top chronic condition (OR= 0.64; 95%CI= 0.56–0.74).

Table 3 provides associations between race, ethnicity and nativity and *each specific* HIT use. Statistically significant associations were found for looking up health information online and refilling prescriptions. In the unadjusted model, foreign-born adults of MENA descent had 18% lower odds (95%CI=0.71–0.95) of looking up health information online compared to US-born White adults. Results remained statistically significant after adjusting for age, sex, highest level of education, and any top chronic conditions (OR=0.67; 95%CI=0.58–0.78). Foreign-born adults of MENA descent had 49% lower odds (95%CI= 0.34, 0.76) of filling a prescription online in the past 12 months compared to US-born White adults. Results remained statistically significant after adjusting for age, sex, highest level of education, and any top chronic conditions (OR= 0.54; 95%CI= 0.40–0.73). There was a statistically significant difference between foreign-born adults of MENA descent and US-born White adults for scheduling medical appointment online in the past 12 months (OR=1.55; 95%CI= 1.15–2.08) in the unadjusted model. However, results were attenuated and no longer significant in the adjusted model. There was not a statistically significant difference (OR= 1.24; 95%CI= 0.93–1.65) in communicating with a healthcare provider by email.

Table 4 provides associations between race, ethnicity and nativity and *all* HIT uses. There were no statistically significant differences between foreign-born adults of MENA descent and US-born White adults in the unadjusted (OR=0.86; 95%CI=0.54–1.36) or multivariable (OR=0.92; 95%CI=0.58–1.46) model.

## DISCUSSION

The purpose of this study was to estimate and compare the prevalence of HIT use among foreign-born adults of MENA descent to US- and foreign-born White adults. An overview of our key findings is presented below.

We found that foreign-born adults of MENA descent were less likely to report *any* HIT use compared to other groups. However, there was no difference in *all* HIT uses compared to US- and foreign-born White adults. We cannot compare these findings to other studies with MENA populations in the US. Furthermore, there is limited research on the patterns of HIT use among adults of MENA descent living in their geographic region of origin. Adults from the MENA region are heterogeneous in many factors, including their internet use. Internet use has been reported to be as high as 99% among residents in Kuwait and Qatar but much lower in Palestine (70.6%) and Jordan (66.8%).^37^ Data from the 2019 US Census indicated that large populations of MENA immigrants in the US are from Iraq (20.7%) and Egypt (17.1%).^38^ Internet use in these countries has been reported as 76% and 58%, respectively, with differences by age, gender, and income within country of origin.^39^ For instance, among older ages (ages 60+ years), HIT use has been reported as only 41% in Iraq and as low as 9% in Egypt. With 26% of our sample of MENA adults ages 60+ years, this may have contributed to our finding that any HIT use was lower than White adults. The research specific to HIT use in the MENA region is limited. Most studies have described recommendations for creating systems for electronic medical records and mobile applications and fail to account for the perspectives of individual users.^40,41^ Two studies in Lebanon evaluated patient perceptions of the usefulness of patient-portals for communication with healthcare providers. Results indicated that patients supported its use for scheduling and communication with healthcare providers, yet no comparisons were made to other populations.^41^ In our study, the prevalence of HIT use for scheduling appointments and communicating with healthcare provider by e-mail was higher among MENA adults than White adults. MENA adults were more likely to use the internet to schedule appointments than US-born White adults in the unadjusted regression model; yet findings were no longer significant after adjusting for age, sex, education, and chronic conditions.

When we looked at each HIT use specifically, foreign-born MENA adults were less likely to look up information online and refill prescriptions. Some reasons for this disparity could be language and underuse of prescription medications among the MENA population. Although English is the most widely spoken language in the US, the incidence of bilingual households is increasing.^42^ Recent data from the US Census indicates that Spanish is the most widely spoken language other than English. Between 2006–2010 and 2017–2021, the greatest increase in language spoken at home other than English was Arabic at roughly a 70% increase.^42^ Although several reputable websites with health information in the US are available in Spanish, fewer are available in Arabic or other languages representing countries in the MENA region. The CDC offers several multilingual health educational printouts on emergency preparedness, health conditions, healthy living, and others. Of the 1,030 health educational printouts available there were 48 only available in Arabic, 14 in Somali and only one in Turkish.^43^ Although website services are available to translate (e.g., Google Translate), heterogeneity in culture, context and linguistic dialects are often not captured with standard electronic-based translation services. We also found that foreign-born MENA adults had 0.54 times lower odds of filling prescriptions online compared US-born White adults. Studies have reported that MENA individuals are less likely to use prescriptions, which may be due to lack of access, barriers in communication with healthcare providers due to language, or cost.^44–46^ Since younger adults are more likely than older adults to report HIT use in general, there may not be a need for refilling prescriptions online among those who are healthy and do not need everyday prescriptions. Yang and colleagues found that adults ages 18–24 less likely to need prescriptions than adults ages 75 and older who were less likely to use the internet for any purpose.^47^

These findings underscore the need for research to examine HIT use among MENA populations both in the US and abroad. Overall, we found that the disparities in HIT use among MENA adults compared to White adults are similar to patterns among other racial/ethnic minority groups (Black, Hispanic) and White adults.^8,9,47^ Without an ethnic identifier for MENA Americans, baseline estimates for all health topics cannot be determined. In 2023, the Office of Management and Budget published a proposal to include a separate checkbox for MENA individuals on the 2030 decennial census and other required federal forms.^48^ A corresponding comment period was opened for 90 days so that members of the public could indicate whether they supported the addition of the checkbox. During the first month of the comment period, 71.49% of all comments posted mentioned the addition of a MENA checkbox and of those, 98.89% indicated support for including it.^49^ The addition of the checkbox will not only allow for baseline health data to be collected for this population, but it will also allow for funding opportunities for linguistic services to be provided. Among the comments reviewed, 55% mentioned a need for a MENA checkbox to support language and linguistic services.^50^ The ability to provide resources for linguistic services is important to developing culturally tailored health education materials and ultimately increasing the ease of access for more HIT use among MENA Americans.

### Strength and Limitations

Despite not being able to describe HIT use among all MENA adults in the US due to the lack of an ethnic identifier, a strength of this study was the ability to combine multiple years of nationally representative data to begin to uncover the patterns of HIT use among the foreign-born MENA population. This method has been extensively to evaluate health behaviors and chronic condition patterns among the foreign-born Middle Eastern population, and more recently expanded to include the North African population. It is important to acknowledge that the results are limited to foreign-born MENA adults only since we are unable to disaggregate US-born MENA adults from the White category. Another strength of the study was the ability to evaluate several HIT uses from the adult sample. The NHIS pilot tested the HIT use set of questions in 2009 and incorporated it as part of its annual question core in 2011. The original set of questions included a question on HIT use for communication with people using chat groups. However, this question was removed in 2018. The question was part of a supplemental set of questions sponsored by the Assistant Secretary for Planning and Evaluation to Health and Human Services to address provisions of the Affordable Care Act of 2010.^51^ This limited our assessment of HIT use for communication purposes to only its use for communication with healthcare professionals. The questions on HIT use did not assess the purpose of and frequency of HIT use. Future studies can expand on these questions to get a broader assessment of HIT uses among this population. The data were cross-sectional. Because of the small samples of foreign-born MENA adults surveyed each year, we combined 8 years of data to provide robust estimates of HIT use in a cross-sectional manner. This method has been used previously to examine MENA health;^19,21^ however, it limits our ability to access trends in HIT use to ensure adequate power with our sample sizes.

## NEW CONTRIBUTION TO THE LITERATURE

In summary, this is one of the first studies to evaluate HIT use among MENA Americans. This study makes an important contribution to the research literature by adding MENA Americans to the discourse on racial/ethnic health disparities in HIT use. Our results highlight that patterns of HIT use among MENA Americans are more similar to other racial/ethnic minority groups than White adults. By masking MENA Americans under the White classification, the health information seeking behaviors cannot be determined. Without an ethnic identifier, this group will remain unnoticed and eHealth efforts for primary, secondary, and tertiary prevention among this population will continue to be omitted.

## Figures and Tables

**Table 1:** Weighted sociodemographic and health information technology (HIT) use characteristics, NHIS 2011–2018, n = 161,613.

	US-born	Foreign-born		
	White n = 155,833 *Column %*	White n = 4,516 *Column %*	MENA n = 1,264 *Column %*	p-value
*Sociodemographic Characteristics*				
Age				.0001
18–44 years	41.6	38.3	50.1	
45–64 years	36.8	34.9	35.8	
65 +years	21.6	26.7	14.1	
Sex				.0111
Male	48.5	45.6	54.1	
Female	51.5	54.4	45.9	
Highest level of education				< .0001
Less than high school	8.9	8.9	8.6	
High school diploma/GED	26.2	20.3	18.3	
Some college/Associates	32.5	28.5	22.8	
Bachelor’s degree or higher	32.5	42.3	50.2	
*Top Chronic Conditions*				
Any Chronic Conditions *(%yes)*	31.66	23.83	20.99	< .0001
Diabetes *(%yes)*	9.02	7.31	8.44	.0089
Coronary heart disease *(%yes)*	5.28	5.47	3.72	.0200
Cancer *(%yes)*	11.59	9.70	5.04	< .0001
Asthma *(%yes)*	13.54	7.26	7.62	<.0001
*Health Information Technology (HIT) Use*				
Any HIT Use *(%yes)*	53.6	50.3	48.9	.0102
Looked up health information on the internet *(%yes)*	51.2	47.8	46.4	.0079
Fill a prescription *(%yes)*	9.0	6.8	4.8	< .0001
Schedule appointment with health care provider *(%yes)*	6.8	7.4	10.2	.0274
Communicate with health care provider by email *(%yes)*	8.6	8.1	10.4	.2358
All HIT Uses *(%yes)*	2.49	2.23	2.89	.5393

Note. All US- and foreign-born groups are non-Hispanic.

**Table 2.** Unadjusted and multivariable associations between race, ethnicity, and nativity status and any health information technology use, NHIS 2011–2018, n= 161,613.

	Any Health Information Technology (HIT) Use	
	Model 1^a^ *OR (95% CI)*	Model 2^b^ *OR (95% CI)*
US-Born *(reference)*		
White	1.00	1.00
Foreign-born		
White	0.89 (0.81–0.96)	0.79 (0.72–0.87)
MENA	0.83 (0.70–0.92)	0.64 (0.56–0.74)

Note. US- and foreign-born groups are non-Hispanic.

Abbreviations. CI=confidence interval; MENA=Middle Eastern and North African; OR=odds ratio.

a Unadjusted model

b Multivariable model adjusted for age, sex, and highest level of education, any top chronic conditions.

**Table 3.** Unadjusted and multivariable associations between race, ethnicity, and nativity status and each specific health information technology use, NHIS 2011 –2018, n= 161,613.

	Look up health information online		Fill a prescription	
	Model 1^a^ *OR (95% CI)*	Model 2 ^b^ *OR (95% CI)*	Model 1 ^a^ *OR (95% CI)*	Model 2 ^b^ *OR (95% CI)*
US-Born *(reference)*				
White	1.00	1.00	1.00	1.00
Foreign-born				
White	0.88 (0.82–0.96)	0.80 (0.73–0.87)	0.74 (0.61–0.90)	0.74 (0.65–0.85)
MENA	0.82 (0.71–0.95)	0.67 (0.58–0.78)	0.51 (0.34–0.76)	0.54 (0.40–0.73)
	Schedule–appointments		Communicate with health care provider by email	
	Model 1 ^a^ *OR (95% CI)*	Model 2 ^b^ *OR (95% CI)*	Model 1 ^a^ *OR (95% CI)*	Model 2 ^b^ *OR (95% CI)*
US-Born *(reference)*				
White	1.00	1.00	1.00	1.00
Foreign-born				
White	1.10 (0.91–1.32)	1.08 (0.95–1.23)	0.94 (0.78–1.13)	0.91 (0.80–1.03)
MENA	1.55 (1.15–2.08)	1.07 (0.86–1.34)	1.24 (0.93–1.65)	1.07 (0.87–1.31)

Note. US- and foreign-born groups are non-Hispanic.

a Unadjusted model

b Multivariable model adjusted for age, sex, and highest level of education, any top chronic conditions.

**Table 4.** Unadjusted and multivariable associations between race, ethnicity, and nativity status and all health information technology uses, NHIS 2011–2018, n= 161,613.

	All Health Information Technology (HIT) Uses	
	Model 1 ^a^ *OR (95% CI)*	Model 2^b^ *OR (95% CI)*
US-Born *(reference)*		
White	1.00	1.00
Foreign-born		
White	0.77 (0.46–1.30)	0.78 (0.46–1.31)
MENA	0.86 (0.54–1.36)	0.92 (0.58–1.46)

Note. US- and foreign-born groups are non-Hispanic.

Abbreviations. CI=confidence interval; MENA=Middle Eastern and North African; OR=odds ratio.

a Unadjusted model

b Multivariable model adjusted for age, sex, and highest level of education, any top chronic conditions.

## Data Availability

Data for this project are publicly available online from the National Center of Statistics (https://www.cdc.gov/nchs/nhis/1997-2018.htm).
